# The Multimodal Ultrasound Features of Ovarian Serous Surface Papillary Borderline Tumor

**DOI:** 10.1089/whr.2021.0140

**Published:** 2022-05-10

**Authors:** Lijun Xie, Xinxiu Liu, Haiying Li, Liyan Huang, Fang Chen, Xingfu Wang, Lei Jiang, Ling Gan

**Affiliations:** ^1^ Department of Medical Ultrasound, The First Affiliated Hospital of Fujian Medical University, Fuzhou, China.; ^2^ Department of Medical Pathology, The First Affiliated Hospital of Fujian Medical University, Fuzhou, China.; ^3^ Department of Hepatobiliary Surgery and Fujian Institute of Hepatobiliary Surgery, Fujian Medical University Union Hospital, Fujian Medical University Cancer Center, Fuzhou, China.

**Keywords:** contrast-enhanced sonography, multimodal ultrasound, ovary, serous surface papillary borderline tumor

## Abstract

**Aim:**

Ovarian serous surface papillary borderline tumor (OSSPBT) is very rare. Combined with clinical and pathological features, we aim to investigate the multimodal ultrasound features of OSSPBT.

**Patients and Methods:**

There were only 18 patients diagnosed with OSSPBT among the 142 patients who were diagnosed with borderline serous ovarian tumor by pathology from June 2008 to December 2020 in our hospital. Their clinical data, conventional ultrasound, two-dimensional contrast-enhanced ultrasound (2D-CEUS), three-dimensional contrast-enhanced ultrasound (3D-CEUS) characteristics, pathology, and prognosis were retrospectively analyzed.

**Results:**

The 18 patients had no specific clinical symptoms. Multiple implantable nodules were found in 8 patients (44.4%), ascites in 13 patients (72.2%), and elevated carbohydrate antigen 125 (CA125) in 15 patients (83.3%). After excluding 2 misdiagnosed patients from 18 patients, 26 tumors in 16 patients (6 unilateral and 10 bilateral) were studied. Conventional ultrasound findings of OSSPBT showed that large solid masses around normal ovary without capsule, and numerous small dense anechoic areas were observed in the parenchyma of the lesion, with strong speckle echo (“blizzard” sign) of varying degrees. The 2D-CEUS and 3D-CEUS showed a normal ovary in the center surrounded by a radial blood supply of OSSPBT with thick and irregular branches. Histopathologically, the papillary fibrous stalk of OSSPBT had a large number of sand bodies and tortuous dilated microvessels. All patients had no recurrence after surgery, and two of them delivered successfully through assisted reproductive technology.

**Conclusion:**

OSSPBT has a good prognosis. Its conventional ultrasound is characterized by irregular solid masses surrounding normal ovaries and a large number of “blizzard” signs. It showed low enhancement of eccentricity with irregular radial branches centered on the ovary by CEUS.

## Introduction

The incidence of borderline ovarian tumors (BOTs) accounts for about 15%–20% of epithelial ovarian tumors.^1^ Its histological characteristics were defined as atypical hyperplasia of the epithelial ovarian cells without destructive stromal infiltration.^2^ The incidence of BOTs appeared 10 to 15 years earlier than epithelial ovarian cancer even with celiac spread or lymph node involvement, but its prognosis is significantly better with 5- and 10-year survival rates reported to be 95% and 85%, respectively_._^1,3,4^ BOTs can be divided into serous BOTs (SBOTs), mucinous BOTs (MBOTs), and borderline endometrioid tumors.

MBOTs are also divided into intestinal type and endocervical type. Fruscella et al. found that SBOTs and endocervical-type MBOTs had very similar sonographic features with a smaller diameter, fewer locules (usually unilocular solid lesions), and a higher color score than intestinal-type MBOT, which was characterized by a significantly higher percentage of lesions with >10 locules.^5^ The study of 64 patients of SBOT by Moro et al. found that according to the International Federation of Gynecology and Obstetrics (FIGO) classification scheme, the vast majority of SBOTs (82.3%) were at stage I, and papillary projections were the most typical ultrasound feature of SBOTs.^6^

Ovarian serous surface papillary borderline tumor (OSSPBT) is a special subtype of SBOT, which is rare to be met in clinic. Previous reports were mostly limited to individual cases.^7^ The pathological features, biological behavior, and prognosis of OSSPBT are between benign serous tumor and low-grade serous carcinoma. Different from typical ultrasound features of SBOT papillary process, OSSPBT presents as a large solid mass, which was often accompanied by abdominal spread and ascites.

By doing so, it is easily misdiagnosed as malignant tumor before surgery. The ultrasound examination of five premenopausal women diagnosed with OSSPBT by Ludovisi et al. found that the affected ovaries were generally normal, but some or all of them were covered by irregular solid tumors.^8^ In addition, OSSPBT tends to occur in women of childbearing age. For young patients with fertility requirements, fertility-preserving surgery can be used to guarantee a higher pregnancy rate.^9^ Therefore, preoperative ultrasound for qualitative diagnosis of OSSPBT is of great significance for the selection of surgical methods and treatment.

The occurrence and metastasis of tumors are closely related to the formation of tumor blood vessels. Abnormal blood perfusion often occurs in tumors. ^10^Contrast-enhanced ultrasound (CEUS) is a noninvasive imaging method to evaluate blood flow perfusion function that has been gradually applied to the ultrasound perfusion imaging of ovarian tumors in recent years.

The diameter of contrast agent microbubbles is similar to that of red blood cells. At low mechanical index, microbubbles can generate strong backscatter signals in tumor microvessels and dynamically obtain tumor microvascular perfusion in real time that not only overcomes the limitation of color doppler flow imaging (CDFI), which cannot display low-velocity blood flow in tumor microvessels,^11^ but also improves the qualitative diagnosis and quantitative evaluation of ovarian tumors. Two-dimensional CEUS (2D-CEUS) can display the perfusion behavior of the lesion in the region of interest at a single level, whereas three-dimensional CEUS (3D-CEUS), which combines CEUS with 3D imaging technology can further display the overall perfusion of the tumor in a stereoscopic, intuitive, and continuous manner.^12^

There are few imaging reports on OSSPBT, and the characteristics of OSSPBT under multimodal ultrasound that combined with conventional ultrasound, 2D-CEUS, and 3D-CEUS have not been reported. In this study, 18 patients of OSSPBT confirmed by pathology in our hospital were retrospectively analyzed. Excluding 2 misdiagnosed patients from 18 patients, 26 tumors in 16 patients (6 unilateral and 10 bilateral) were studied. Combined with its clinicopathological features, the diagnostic value of multimodal ultrasound in OSSPBT will be discussed in our study.

## Patients and Methods

### Clinical data collection

From June 2008 to December 2020, 142 patients of ovarian borderline tumor confirmed by pathology were treated in our hospital, including 18 cases of OSSPBT. Clinical, laboratory, and preoperative conventional ultrasound data of these 18 patients were collected, their tumor pathological sections were evaluated by pathologists, and FIGO staging of lesions was determined.^13^ Two of the 18 OSSPBT patients, a 38-week gestation and a woman with ovarian teratoma, were misdiagnosed by ultrasound, so only 16 patients with a total of 26 ovarian lesions (6 unilateral and 10 bilateral) had further CEUS.

The CEUS findings were compared with those of the 26 OSSPBT synchronous normal myometriums, 30 patients with benign ovarian serous cystadenoma (BOSC), and 30 patients with malignant ovarian serous cystadenocarcinoma (MOSC) confirmed by pathology. This study was approved by the institutional Ethics Committee of our hospital, and the informed consent was signed by all participants.

### Ultrasonic instruments and methods

#### Conventional ultrasonic scanning

The equipment routinely used in the study was an IU22 US system (Philips Medical Systems, Bothell, WA) with a convex array probe (C5-1, 1–5 MHz) and an intracavitary probe (3D9-3v, 3–9 MHz). The pelvic viscera were examined by routine abdominal and vaginal scanning to observe the location, size, echo, and blood supply of the uterus and bilateral ovaries. If abnormal masses were found, their location, size, echo, and blood supply were observed and particular attention was paid to their relationship with the ovaries, mass diffusion in the pelvis, peritoneum, and abdominal pelvic effusion.

Subjective semiquantitative evaluation of tumor blood supply was conducted according to the International Ovarian Tumor Analysis rules (color score 1: no blood flow signal; color score 2: only a small amount of blood flow was detected; color score 3: moderate amount of blood flow; color score 4: abundant blood flow signal).^14^

#### Contrast-enhanced ultrasound

SonoVue (BRACCO company, Italy) was used as contrast medium. The suspension was made by dissolving and diluting with a 5 mL of saline solution and shaking. None of the 16 patients had severe renal insufficiency, pulmonary insufficiency, or cardiac insufficiency. In compliance with ethical factors, they signed an informed consent form for CEUS examination and 1.5 mL SonoVue suspension was injected through the central cubital vein by mass injection, followed by rapid injection of 5 mL normal saline into the tube.

After selecting the best observation section of the patient's tumor and displaying the same patient's normal myometrium of the uterus as the contrast reference, fix the probe position, and enter the contrast state. A contrast agent was injected and a CEUS timer was started at the same time to continuously observe the contrast process of tumor and retain dynamic images. Observe the perfusion characteristics of the mass until the contrast agent was completely dissipated. The enhanced ultrasound examination would take at least 5 minutes. Using Q-Lab company, USA (QLAB) quantitative analysis software (Philips Medical version 9.1) automatically generates the contrast time–intensity curve (TIC) of the lesions.

The real part of the lesions with the same area and the normal uterine myometrium with the same area are selected as region of interest. The distribution characteristics of the imaging curves in the interested areas are analyzed. The following imaging parameters are recorded: the rise time (RT), time to peak (TTP), peak intensity (PI), area under the curve (AUC), half time of descending peak intensity (HT), mean channel time (MCT), and wash in slope (WIS).

After the contrast agent was completely cleared, the sampling frame size and scanning Angle (85°) in the area of interest were adjusted. The abdominal 3D volume probe was enabled to enter the 3D imaging mode and 1.2 mL acoustic contrast agent was injected intravenously in the same way during 2D-CEUS to observe the angiography process of the tumor. The time to start 3D angiography was determined according to the 2D venography of tumors. Also, the characteristics of tumor perfusion and nourishing vessels in arterial phase were observed and the images were stored. The 3D volumetric imaging software built into the instrument was used to analyze the spatial distribution characteristics of tumor vessels.

QLAB quantitative analysis software is necessary in this project research. If without it, the original ultrasound images and data cannot be analyzed, real-time microcirculation perfusion imaging of tumors also fail to be quantitatively analyzed, and further differential diagnosis of ovarian tumors can be affected.

In this study, five authors are engaged in ultrasonic scanning and ultrasonic diagnosis report. The ultrasonic report was obtained by analyzing the patient's clinical data and original ultrasonic images. Two of the authors are doctors with senior professional titles in ultrasound and have been engaged in gynecological clinical ultrasound diagnosis for >20 years. The other three ultrasound doctors have >10 years of gynecological clinical ultrasound diagnosis experience.

Ultrasonic scanning and ultrasonic diagnosis were completed by the same doctor. In case of difficult cases, it is necessary to consult gynecological ultrasound experts with senior professional titles to discuss the ultrasound report and obtain consensus results. Additionally, one of the authors is a pathologist with senior professional title, who has nearly 20 years of working experience in gynecological pathological diagnosis.

### Statistical analyses

The statistical analyses were performed by using SPSS version 23.0 (SPSS, Inc., Chicago, IL). The counting data were expressed as percentage, and the measurement data were expressed as mean ± standard deviation ($\bar{x}\pm s$). Data of each group were tested for homogeneity of variance and normality. The mean value of each group was compared by Student Newman Keuls-*q* test, and *p* < 0.05 was considered as statistically significant.

## Results

### Clinical features

The clinical data of the 18 patients are shown in Table 1. The average age of 18 OSSPBT patients was about 34 years of age (range 22–48 years). Among the 18 patients of OSSPBT, 8 patients (44.4%) were unilateral and 10 patients (55.6%) were bilateral. All patients did not have any specific clinical symptoms. Pelvic mass was found in twelve patients (66.7%) during physical examination, 4 patients (22.2%) were diagnosed with abdominal pain for >1 month, and ovarian mass was found in 2 patients (11.1%) during cesarean section. Multiple implantable nodules were found in 8 patients (44.4%) and ascites in 13 patients (72.2%). Carbohydrate antigen 125 (CA125) was elevated in seven cases (77.8%), while carbohydrate antigen 19-9 (CA19-9), CEA was in normal range for all patients.

**Table 1.** Clinical and Pathological Features' Analysis of Ovarian Serous Surface Papillary Borderline Tumor (18 Patients)

Parameter	***n*** (%)
Clinical features	
Elevated serum CA 125	15/18 (83.3%)
Affected side	
Bilateral	10/18 (55.6%)
Unilateral	8/18 (44.4%)
Pelvic implant nodules	12/18 (66.7%)
Ascites	13/18 (72.2%)
Surgical treatment	
Radical operation	10/18 (55.6%)
Tumor exfoliation	5/18 (27.8%)
Adnexectomy	3/18 (16.6%)
Tumor recurrence or metastasis on follow-up	0/18 (0%)
Pathological features (FIGO stage)	
Stage I	10/18 (55.6%)
Stage II	2/18 (11.1%)
Stage III	6/18 (33.3%)

CA125, carbohydrate antigen 125; FIGO, International Federation of Obstetrics and Gynecology surgery; OSSPBT, ovarian serous surface papillary borderline tumor.

Among the 18 patients, 10 patients (55.6%) underwent radical operation, 5 patients (27.8%) underwent exfoliation of ovarian masses, and 3 patients (16.6%) underwent adnexectomy. There was no tumor recurrence in all patients during follow-up. Two of them were successfully pregnant and delivered by assisted reproductive technology after exfoliation of ovarian masses.

### Conventional ultrasonic characteristics

**FIG. 1.**
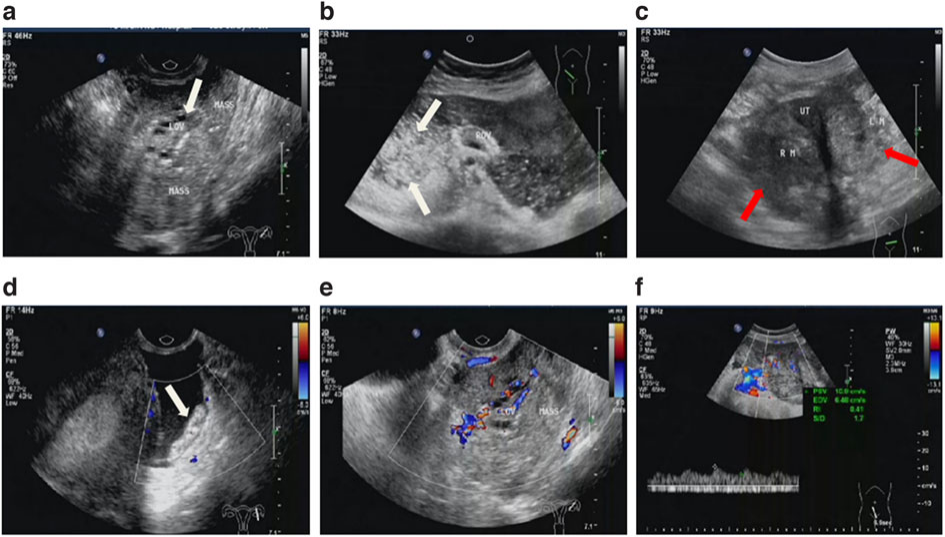
Conventional ultrasonic characteristics of OSSPBT. **(a)** A large area of solid echo was seen around the ovary, and a normal ovary with small follicles (white arrow) was seen in the mass. **(b)** The ovary is surrounded by a large number of solid mixed echoes, and a large number of speckled strong echoes are seen, showing “blizzard” sign (white arrow). **(c)** MOSC was bilateral ovarian irregular solid or mixed echo mass (red arrow), no normal ovarian tissue echo. **(d)** There were tumor planting nodules (white arrow) and punctate blood flow signals in the retroperitoneum of the pelvic cavity. **(e, f)** CDFI showed mild or moderate blood flow signals in the tumor, PSV: 10.9 cm/s, RI: 0.41, and color score: 3. CDFI, color doppler flow imaging; LM, left ovarian lesion; Mass, lesions; MOSC, malignant ovarian serous cystadenocarcinoma; OV, ovary; PSV, peak systolic velocity; RI, resistance index; RM, right ovarian lesion; UT, uterus.

Two patients were misdiagnosed by ultrasound from 18 patients of OSSPBT, one of whom was a 38-week pregnant woman and another was someone with ovarian teratoma, respectively, so 26 tumors in remaining 16 patients (6 unilateral and 10 bilateral) were studied. The maximum diameter of the tumor was about 132 mm. Eight patients had multiple similar implanted nodules on the surface of uterus, retroperitoneum, or greater omentum (Fig. 1d).

The conventional ultrasound findings of all tumors were that irregular solid echo mass close to or wrapped around the ipsilateral ovary without obvious capsule, and normal ovary with small follicles could be seen in the mass (Fig. 1a, b). But MOSC was an irregular solid or mixed echo mass in the ovarian adnexal area, without normal ovarian tissue echo (Fig. 1c).

In 14 patients of OSSPBT, there were many dense small anechoic areas in the tumor parenchyma, presenting a fine grid change. There were also different degrees of speckled strong echo areas in the mass, which were mixed with dense small anechoic areas, showing a “blizzard” sign (Fig. 1b). However, the parenchyma of the mass was isoechoic in four patients, where only a small part of the mass had dense and small anechoic areas, with no obvious punctate hyperecho.

There were mild or moderate blood flow signals in OSSPBT (Fig. 1e, f). The color score ranged from 2 to 3.

### Features of CEUS

**FIG. 2.**
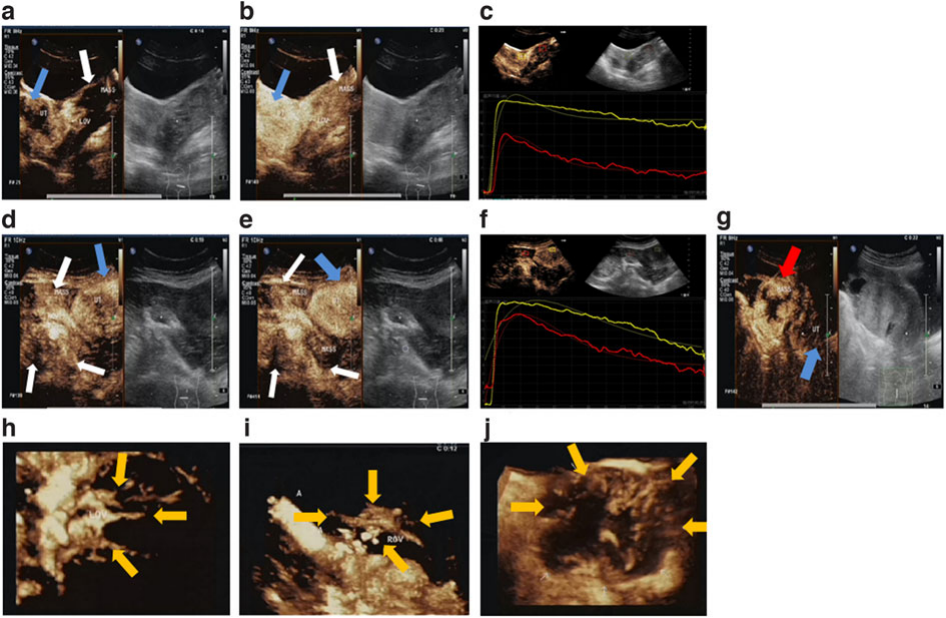
CEUS features of OSSPBT. **(a–c)** 2D-CEUS: the mass (white arrow) around the left ovary began to strengthen later than the uterine wall (blue arrow) **(a)**, and the PI of the mass (white arrow) was lower than that of the uterine wall (blue arrow) **(b)**. The contrast TIC curve of the lesion and myometrium **(c)** red is the lesion, yellow is the myometrium. **(d–f)** 2D-CEUS: the mass (white arrows) around the right ovary and the uterine wall (blue arrows) were in a synchronous low enhancement **(d)**, and the regression rate was faster than that of the uterine wall (blue arrows) **(e)**. The contrast TIC curve of the lesion and myometrium **(f)** red is the lesion, yellow is the myometrium. **(g)** The 2D-CEUS of MOSC (red arrow) showed that the centripetal enhancement was earlier than that of uterine wall (blue arrow). **(h, i)** 3D-CEUS: the trophoblastic vessels of the tumor originate from the left ovary and are radial branches (yellow arrows) **(h)**. The tumor is supplied by large branching blood vessels around the right ovary (yellow arrows) **(i)**. **(j)** The 3D-CEUS of MOSC showed irregular branches of nutrient vessels (yellow arrows) from the periphery to the center. 2D, two-dimensional; 3D, three-dimensional; CEUS, contrast-enhanced ultrasound; PI, peak intensity; TIC, time–intensity curve.

Two-dimensional-CEUS and 3D-CEUS were performed in 26 tumors of the 16 OSSPBT patients. On 2D-CEUS, the main nutrient vessels of OSSPBT were observed to mainly emit from the periphery of the affected ovary. OSSPBT imaging was performed in a manner of discrete heterogeneous enhancement that started more slowly than the uterine wall (66.7%, Fig. 2a, c) or synchronously with the uterine wall (33.3%, Fig. 2d, f), and their angiographic regression was faster than the uterine wall (Fig. 2b, e), while the contrast-enhanced ultrasonography of MOSC showed a high enhancement of centripetal nonuniformity earlier than the uterine wall (Fig. 2g).

On 3D-CEUS, the nutrient vessels of OSSPBT radiate irregularly from the periphery of the ovary (Fig. 2h, i), whereas the nutrient vessels of MOSC branch irregularly from the periphery of the tumor into the tumor (Fig. 2j). The TIC of tumor was analyzed quantitatively (Table 2). The results are as follows. Compared with OSSPBT synchronous myometrium, the WIS, TTP, PI, and HT of OSSPBT were significantly decreased (*p* < 0.05). Compared with BOSC, the TTP of blood perfusion in OSSPBT was faster, the AUC increased, and the HT prolonged (*p* < 0.05). Compared with MOSC, the PI of blood perfusion and WIS of OSSPBT decreased (*p* < 0.05).

**Table 2.** Comparison of Time–Intensity Curve Quantitative Parameters Among Ovarian Serous Surface Papillary Borderline Tumor (26 Tumors in 18 Patients), Ovarian Serous Surface Papillary Borderline Tumor Synchronous Myometrium, Benign Ovarian Serous Cystadenoma, and Malignant Ovarian Serous Cystadenocarcinoma

***Groups***	***N***	RT (seconds)	TTP (seconds)	PI (dB)	AUC (dB·s)	HT (seconds)	MCT (seconds)	WIS (dB/s)
OSSPBT	26	15.21 ± 1.31	25.12 ± 1.65^*^^,^^**^	14.25 ± 2.06^*^^,^^***^	1465.34 ± 283.12^**^	77.32 ± 7.54^*^^,^^**^	18.21 ± 2.87	0.74 ± 0.05^*^^,^^***^
Myometrium	26	17.65 ± 1.11	35.32 ± 1.12	23.43 ± 1.54	2192.32 ± 135.43	94.45 ± 4.32	23.67 ± 1.76	1.289 ± 0.21
BOSC	30	14.54 ± 1.22	37.87 ± 3.54	10.46 ± 1.87	654.32 ± 57.59	40.94 ± 2.69	22.65 ± 2.28	0.57 ± 0.78
MOSC	30	15.45 ± 1.98	30.67 ± 2.93	24.74 ± 1.65	2053.54 ± 205.82	67.43 ± 6.49	20.59 ± 1.69	1.80 ± 0.23

^*^ *p* < 0.05, compared with myometrium.

^**^ *p* < 0.05, compared with BOSC.

^***^ *p* < 0.05, compared with MOSC.

AUC, area under the curve; BOSC, benign ovarian serous cystadenoma; HT, half time of descending peak intensity; MCT, mean channel time; MOSC, malignant ovarian serous cystadenocarcinoma; OSSPBT, serous surface papillary borderline tumor; PI, peak intensity; RT, rising time; TIC, contrast time–intensity curve; TTP, time to peak; WIS, wash in slope.

### Pathological findings

**FIG. 3.**
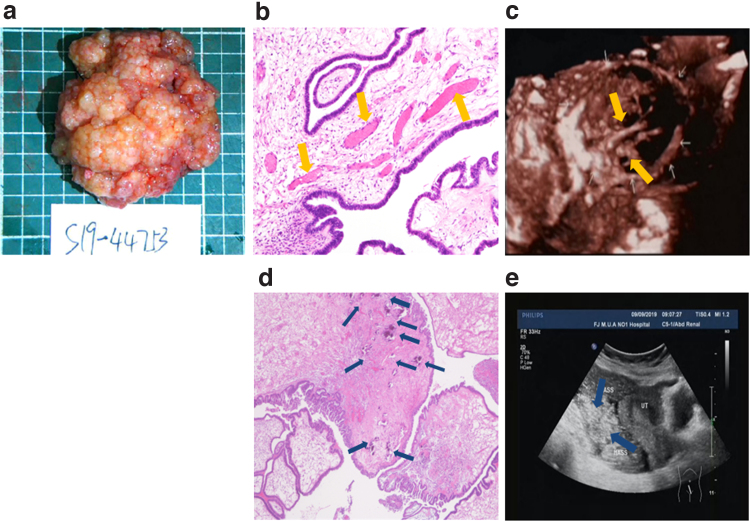
Pathological image of OSSPBT and its corresponding ultrasonic image. **(a)** The gross appearance of the tumor was a large number of white and transparent bead-like nodules fused into a cauliflower pattern. **(b)** HE staining showed the papilla or micropapillary structure of the cells covered with single layer or multilayer cubic to columnar cells, and there were more dilated and congested microvessels (yellow arrows) in the axis of connective tissue ( × 200). **(c)** The irregular branching blood vessels (yellow arrows) were visible in its corresponding 3D ultrasound image. **(d)** HE staining showed that the fiber connective tissue axis saw a large amount of gravel (blue arrows) ( × 200). **(e)** A large number of strong echo speckled echo spot (blue arrows) in the tumor were visible in its corresponding conventional ultrasound image. HE, Hematoxylin–Eosin.

The pathological results of all tumors showed OSSPBT. The gross appearance of the tumors was no capsule, showing a large number of white transparent beads like nodules fused into a cauliflower pattern (Fig. 3a). Among them, 10 patients (60%) showed different degrees of implanted nodules in the pelvic cavity. Microscopically, the tumor cells showed papillary hyperplasia and junctional morphology.

There were many dilated vessels in the axis of fibrous connective tissue in the center of the nipple (Fig. 3b), and different numbers of sand particles were observed (Fig. 3d), which is consistent with the irregular branching blood vessels (Fig. 3c) and a large number of speckled strong echo (“blizzard” sign) (Fig. 3e) in the tumor in the multimodal ultrasound image. No lymph node involvement was found in all patients with OSSPBT.

Postoperative staging, according to the FIGO criteria, was I in 10 (55.6%), II in 2 (11.1%), and III in 6 (33.3%) of the 18 patients (Table 1).

## Discussions

OSSPBT grows on the surface of the ovary without envelop. Our research found that the conventional ultrasound features of OSSPBTs are irregular solid masses surrounding normal ovaries and a large number of “blizzard” signs, which are closely related to pathological sand bodies. Thus, we believe that its characteristic ultrasonic manifestations are closely related to its pathological structure. Gray scale ultrasound showed that there were irregular solid echoes around the normal ovary, and a large number of dense small anechoic areas with dot or linear strong echoes could be seen in the solid echoes. This unique ultrasonic physical characteristic is related to a large number of transparent fish seed nodules with dense distribution in pathology.

Besides, different degrees of speckled strong echo could be seen, and the typical one could show the snowstorm sign. The formation of the speckled strong echo may be closely related to the existence of a large number of sand bodies in the axis of the papillary fibrous stalk of OSSPBT pathologically. Some researchers believed that the presence of tumor sand bodies meant that they had good biological behavior and prognosis.^15^ They could block the growth of tumor cells and even formed a barrier for tumor metastasis.^16^

Different from the invasive implantation of ovarian serous cancer, OSSPBT is prone to diffusion implantation that spread out of the ovary in a noninvasive way that progresses slowly, and has a much higher survival rate than concurrent ovarian cancer.^2^ In this study, all patients with OSSPBT had no tumor recurrence and had a good prognosis. Ultrasound could show sensitively implantable pelvic nodules, especially in the presence of ascites. It should be noted that when there is no or a small amount of pelvic effusion, the mass is disturbed easily by the strong echo of gas in the abdominal pelvic intestinal tube.

In our research process, one of the two misdiagnosis on patients may be caused by the increased uterine and pelvic intestinal gas during pregnancy, which also reminds us that special attention should be paid to the peripheral situation of the ovary in the scan of the adnexal area, and the periovarian lesions can be observed by using the dual-combination method of pressure probe.

The density, morphology, distribution, and function of new microvessels in tumor stroma are the pathological basis of CEUS perfusion imaging.^10,17^ As a blood storage pool development technology, CEUS can display the blood perfusion of ovarian tumors in real-time and quantitatively analyze the information of tumor contrast time–enhancement curve, so as to objectively evaluate the blood perfusion characteristics of tumors.^10–12^

The parameter PI in the contrast curve represents the maximum value of microcirculation perfusion in the region of interest, while the TTP reflects the time when the maximum value of microcirculation perfusion reaches. The WIS is the ascending slope of the curve, which reflects the inflow velocity of microcirculation. The HT is the time taken for the curve intensity to decrease from the peak value to half of the peak value, reflecting the outflow velocity of blood and the AUC is the area under the entire intensity curve. Ordén et al.^18^ found in the study of CEUS on adnexal masses that the AUC and DCE (duration of contrast agent effect) are differences between the benign and malignant groups that help to identify benign and malignant neoplasm.

Yang et al.^19^ found that the perfusion parameters such as PI, HT, and AUC could be used as effective indicators to distinguish benign and malignant ovarian tumors and to distinguish whether ovarian masses were neoplastic lesions. However, CEUS for OSSPBT is seldom reported at present.

In this study, it can be seen that the tumor body is large and solid and the enhancement of OSSPBT was characterized by uneven and low enhancement of eccentricity, synchronous, or later than that of the uterine wall with no obvious perfusion defect. Compared with the benign group, the OSSPBT group had a shorter TTP, an increased AUC, and a longer HT, while PI and WIS decreased in the OSSPBT group compared with the malignant group (*p* < 0.05), indicating that the microvascular perfusion of OSSPBT was between benign serous tumor and ovarian serous cancer.

As a comprehensive staging procedure, fertility preservation surgery is the standard of care for young BOT patients (Grade A Recommendation).^20^ Compared with radical surgery, postoperative recurrence rate will be increased, but there is no significant difference in overall survival rate. The surgical scope of staging surgery, especially ovarian tumor exfoliation, was of great benefit to the postoperative pregnancy success rate.^21^ We can see from the study that two young patients who underwent OSSPBT stripping were successfully pregnant and delivered 1 year later by assisted technology.

The tumor marker CA125 is mainly expressed in ovarian serous tumors, but the level of CA125 can be overlapped between OSSPBT and serous papillary carcinoma.^22^ CA125 combined with ultrasonography can be used to monitor postoperative tumor recurrence in serous borderline tumors.^23^ In this study, 15 patients (83.3%) with OSSPBT showed different levels of serum CA125 increase before operation, but no tumor recurrence was found in regular ultrasound reexamination and serum CA125 level monitoring 1 year after operation. Due to the tendency of long-term recurrence of borderline tumors, long-term rigorous follow-up of >10 years is still needed after operation.

For young BOT patients with fertility requirements, preoperative ultrasound diagnosis and differential diagnosis are very important to evaluate their reproductive ability and surgical management. This study is a single-center preliminary study and has obtained considerable results, but OSSPBT is very rare and limited to fewer patients. It is hoped that multicenter research can be combined to obtain a large number of patients to further confirm our conclusion in the future.

## Conclusion

OSSPBT has a good prognosis. Its conventional ultrasound features are irregular solid masses surrounding normal ovaries and a large number of “blizzard” sign, which are closely related to pathological sand bodies and could be a new sign of BOTs following confirmation in further multicenter prospective studies. CEUS is characterized by centrifugal low enhancement and irregular radial branches, which is closely related to the tortuous and dilated microvessels in pathology. When ovarian tumors show the above characteristics, they should be considered as a diagnosis.

## Abbreviations Used

2D-CEUS: two-dimensional contrast-enhanced ultrasonography
3D-CEUS: three-dimensional contrast-enhanced ultrasonography
AUC: area under the curve
BOSC: benign ovarian serous cystadenoma
BOTs: borderline ovarian tumors
CA19-9: carbohydrate antigen 19-9
CA125: carbohydrate antigen 125
CDFI: the color doppler flow imaging
CEA: carcinoembryonic antigen
FIGO: the international Federation of Obstetrics and Gynecology Surgery
HE: Hematoxylin–Eosin
HT: half time of descending peak intensity
MCT: mean channel time
MOSC: malignant ovarian serous cystadenocarcinoma
OSSPBT: ovarian serous surface papillary borderline tumor
PI: peak intensity
PSV: peak systolic velocity
QLABQ: Q-Lab company, USA
RI: resistance index
RT: Angiographic rising time
TIC: time–intensity curve
TTP: time to peak
WIS: wash in slope
